# Synergistic Effect of Brevetoxin BTX-3 and Ciguatoxin CTX3C in Human Voltage-Gated Na_v_1.6 Sodium Channels

**DOI:** 10.1021/acs.chemrestox.3c00267

**Published:** 2023-11-15

**Authors:** Sandra Raposo-Garcia, Celia Costas, M. Carmen Louzao, Mercedes R. Vieytes, Carmen Vale, Luis M. Botana

**Affiliations:** † Departamento de Farmacología, Farmacia y Tecnología Farmacéutica, Facultad de Veterinaria, IDIS, Universidad de Santiago de Compostela, Campus Universitario s/n, Lugo 27002, Spain; ‡ Departamento de Fisiología, Facultad de Veterinaria, Universidad de Santiago de Compostela, Campus Universitario s/n, Lugo 27002, Spain

## Abstract

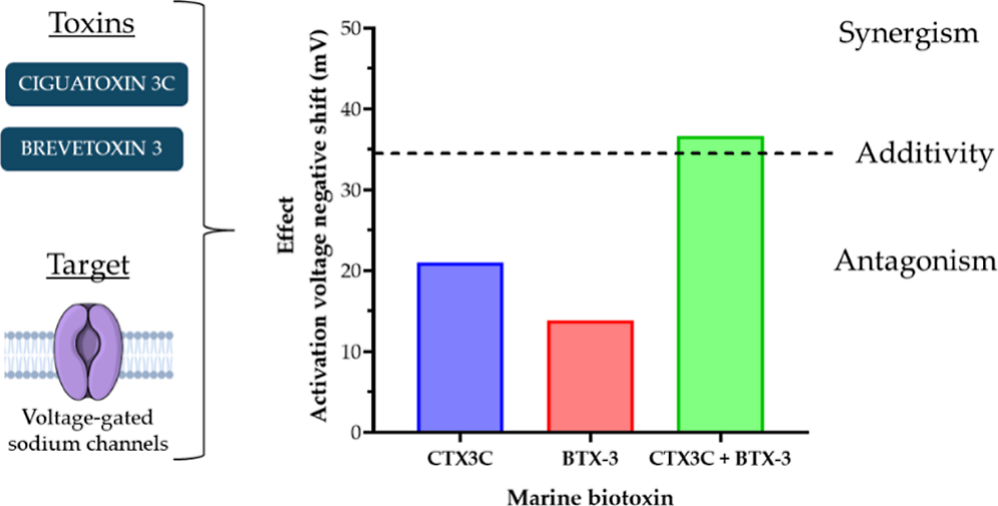

Emerging marine biotoxins such as ciguatoxins and brevetoxins have been widely and independently studied as food pollutants. Their maximum levels in food components were set without considering their possible synergistic effects as consequence of their coexistence in seafood and their action at the same cellular target. The absolute lack of data and regulations of the possible combined effects that both marine biotoxins may have raised the need to analyze their direct *in vitro* effects using electrophysiology techniques. The results presented in this study indicate that ciguatoxins and brevetoxins had a synergistic effect on human Na_v_1.6 voltage-gated sodium channels by hyperpolarizing their activation and inactivation states. The results presented here indicate that brevetoxin 3 (BTX-3) acts as partial agonist of human sodium channels, while ciguatoxin 3C (CTX3C) was a full agonist, explaining the differences in the effect of each toxin in the channel. Therefore, this work sets the cellular basis to further apply this type of studies to other food toxicants that may act synergistically and thus implement the corresponding regulatory limits considering their coexistence and the risks to human and animal health derived from it.

## 1 Introduction

**Figure 1:**
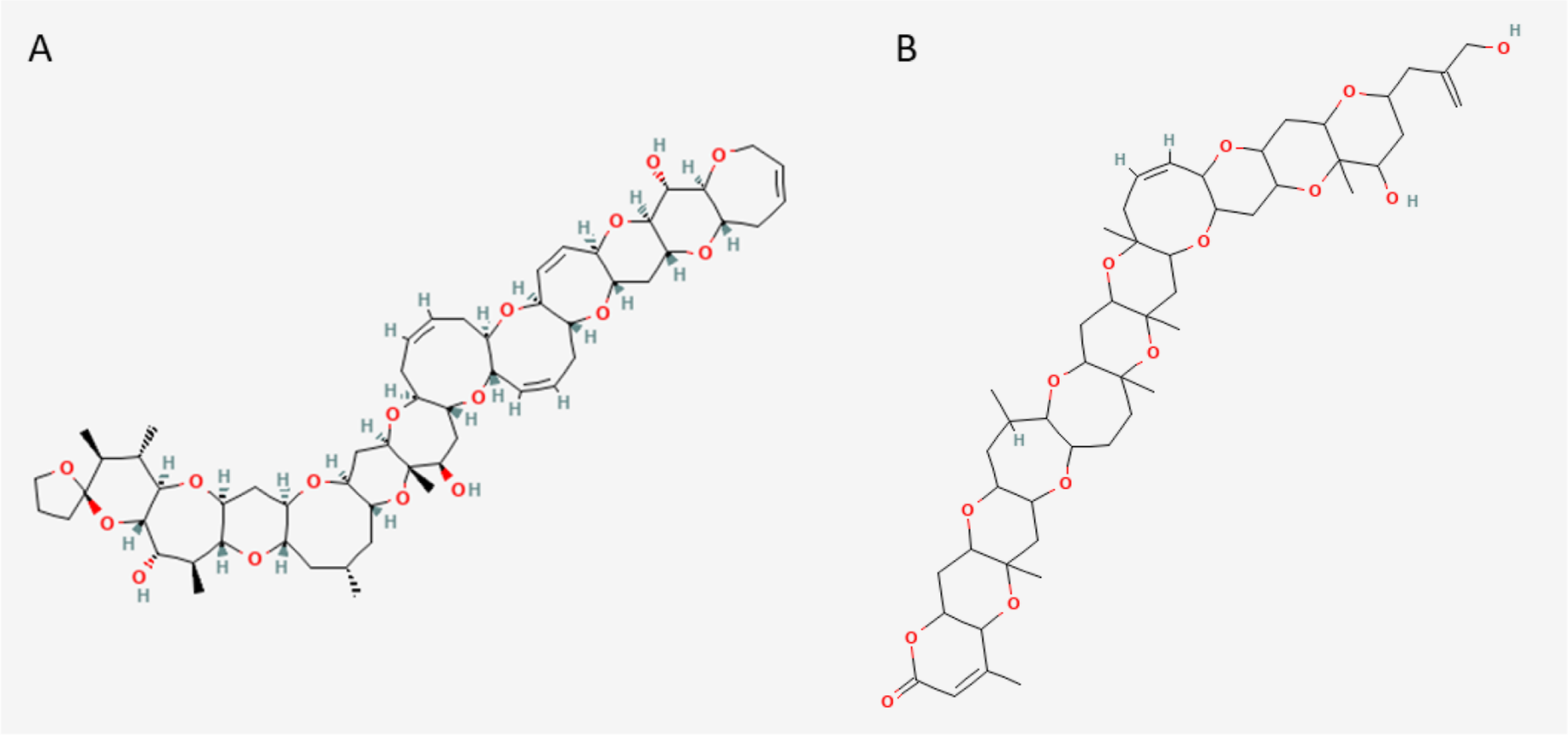
Chemical structure of the studied marine toxins, CTX3C (A) and BTX-3 (B).

Brevetoxins (BTXs), produced naturally by dinoflagellates of the genus *Karenia brevis*, and ciguatoxins (CTXs), produced by *Gambierdiscus* and *Fukuyoa* genera, are cyclic polyether toxins with analogous chemical structures, shown in Figure 1. Both BTXs and CTXs can accumulate along the marine food chain, leading to seafood poisoning in humans and animals. The ingestion of BTX- and CTX-contaminated fish and shellfish results in quite similar symptomatology including gastrointestinal, neurological, and cardiovascular symptoms commonly known as neurotoxic shellfish poisoning (NSP)^1^ and ciguatera poisoning (CP), respectively. However these symptoms are more severe and longer-lasting for CP.^2^ These physiological alterations caused by CTXs and BTXs are a consequence of their effect altering the voltage-gated sodium channels (VGSCs), which are essential transmembrane proteins involved in cellular excitability.^3^ However, despite being similar in their mechanism of action, there are differences in the reported effects and potency between the two groups of toxins that indicate that BTXs act at micromolar concentrations, while CTXs present physiological effects at nanomolar concentrations.^4,5^

CTXs and BTXs bind to a common site of the sodium channel located at the cleft created by segment S5 of domain IV, segment S6 of domain I, and the P-loop (P1) that connects segments S5 and S6 of domain IV of the VGSC α-subunit^6,7^ but with different affinities. Previous studies indicated that CTXs interact specifically and with higher affinity with sodium channels;^8,9^ however, the differences in the chemical structures between the CTX analogues generate modifications in their binding and activity over VGSC.^10,11^ BTXs are also considered to have a high binding affinity to sodium channels;^12,13^ however, the differences between both groups of toxins are significant since studies of their binding affinity to rat brain sodium channels allowed to calculate an inhibitory constant (ki) of 0.041 nM for ciguatoxin-1B which was more than 50-fold higher than that of BTX-1 (2.24 nM).^10^ The difference in binding to VGSC among BTXs and ciguatoxin 3C (CTX3C) is less noticeable since CTX3C has only 20-fold higher affinity (ki = 0.47 nM) than that of BTX-3s, at concentrations of 1 nM of the compounds.^10^ Because of the higher binding affinity of CTXs to VGSC, CTX3C can displace brevetoxin 3 (BTX-3) from their binding at site 5^14^ of the protein.

The functional consequence of the interaction of CTXs and BTXs with VGSC is a hyperpolarizing shift in the sodium channel activation curve^5,7,15^ as well as a negative shift of their half inactivation voltage.^16^ CTXs are known to have a significant effect on the activation and inactivation state of VGSC at concentrations of 1 nM and higher.^7,11,17^ As a consequence, the channels will remain permanently open at resting cell membrane potentials, resulting in depolarization of the membrane and spontaneous and/or repetitive action potential discharges in excitable cells.^6,9^ Although both groups of toxins interact with the same site of VGSC, there are important differences between the observed cellular effect of each group since CTXs cause a remarkable decrease in the maximum peak inward sodium current amplitude (*I*_Na_),^7,11,17^ while no significant effects were reported for BTXs.^4,5^ It is important to note that the effects and the affinity of CTXs or BTXs for VGSC depend on the analogue and the channel subtype.^18^ Moreover, the binding of BTXs to sodium channels is highly state-dependent, thus, channel opening facilitates BTX binding, feeding back their binding and sodium influx.^15^

Due to the danger that marine biotoxins represent for human and animal health, their limits for international trade in fishery products were set more than a decade ago by the Codex Committee on Fish and Fishery Products (CCFFP) (CODEX STAN 292-2008). The levels of biotoxins in live and raw bivalve mollusk flesh were established for different groups of marine toxins taking into account the limits for each biotoxin group.^19^ The maximum levels for BTXs were established as 20 MU (mouse units)/100 g shellfish flesh (800 μg/kg) for BTXs;^19^ however, recent studies have reported that this maximum level does not appear protective enough^20^ based on the previous lowest observed adverse effect level established at 0.3–0.4 MU/kg body weight.^21^ Currently, the American FDA applies a threshold of 800 μg BTX-2 equivalents/kg shellfish flesh and a guidance level for Caribbean CTXs of 0.1 μg/kg C-CTX1 equivalents and 0.01 μg/kg for P-CTX1B.^22^ Although these marine biotoxins constitute an emerging risk in Europe, there are still no regulatory official limits, and only a guidance level of 180 μg BTX-3 eq/kg shellfish meat was proposed by the French Agency for Food, Environmental, and Occupational Health and Safety.^20^

The worldwide spread of emergent marine biotoxins, already affecting European coasts, and the scarce reliable information about their biological effects led to several international organizations such as FAO, WHO, and EFSA highlighting the need for a full re-evaluation of the toxicity and relative potencies of these food contaminants.^23,24^ Moreover, the coexistence of CTXs and BTXs in fishery products has been previously reported.^25−28^ In addition, the possibility of misdiagnosis of NSP with CP has been considered due to their similar symptomatology, and methods to discriminate CTXs from BTXs in fish tissue had been developed.^26^ Both, BTXs and CTXs, can also accumulate in water and air,^29^ increasing the risk of coexposure to both groups of marine biotoxins. So far, the broad reports on the activity of marine toxins evaluated only their separate effects even when they share the same molecular target. The co-occurrence of BTXs and CTXs may represent a potential risk for human health.^30,31^ In this work, the potential risks of the simultaneous presence of both groups of toxins in food based on their effects in the functional activity of human sodium channels have been evaluated for the first time.

## 2 Materials and Methods

### 2.1 Chemicals and Toxins

Pacific CTX CTX3C was purchased from Wako (FUJIFILM Wako Chemicals Europe GmbH, Neuss, Germany, purity 99%) and dissolved in dimethyl sulfoxide (DMSO) at a final concentration of 10 μM. For experiments, 1 μM solutions were made in Lockés buffer containing: 154 mM NaCl, 5.6 mM KCl, 1.3 mM CaCl_2_, 1 mM MgCl_2_, 10 mM *N*-(2-hydroxyethyl)piperazine-N′-ethanesulfonic acid (HEPES), and 5.6 mM glucose. The pH was adjusted to 7.4 with Trizma base. BTX-3 with a 95% purity was purchased from Latoxan (France) and dissolved in ethanol at a final concentration of 50 μM. For experiments, consecutive dilutions were performed in physiological Lockés buffer. The maximum solvent concentration used had no effect on VGSCs. All other chemicals were of reagent grade and purchased from Sigma.

### 2.2 Human Cell Cultures

The human embryonic kidney cell line (HEK293) transfected with the α subunit of the Na_v_1.6 sodium channel isoform was kindly provided, under a material transfer agreement, by GlaxoSmithKline R&D (Stevenage, U.K). The cells were cultured in Dulbecco’s modified Eagle’s medium (DMEM)/F12 medium enriched with glutamax, nonessential amino acid solution (MEM, Gibco, 1% w/v), 10% fetal bovine serum, and 0.4 mg/mL Geneticin (G418, Gibco) and maintained at 37 °C in a humidified 95% O_2_/5% CO_2_ atmosphere until they reached 80% of confluence, replacing the medium every 2 days. The cells were subcultured in 12-well plates in glass coverslips at a density of 60,000 cells/mL. Twenty-four or 48 h before electrophysiological recordings, the cells were placed at 30 °C to maximize sodium channel expression.^32^

### 2.3 Electrophysiological Recordings

For electrophysiological recordings, glass coverslips with cells were placed in a recording chamber with 0.5 mL Lockés buffer as the extracellular solution. Recording electrodes were fabricated with borosilicate glass microcapillaries (1.5 outer diameter) and had resistances ranging from 4 to 10 MΩ. Pipettes were filled with an intracellular solution containing 120 mM CsF, 10 mM EGTA, 10 mM HEPES, and 15 mM NaCl and pH adjusted to 7.25 with CsOH. Voltage-gated sodium currents were recorded at room temperature 5 min after reaching the whole-cell configuration with a Multiclamp 700B amplifier and digitalized with the Digidata 1440A (both from Axon Instruments, California, U.S.A.) maintaining the cells at a holding potential (*V*_hold_) of −55 mV. Signals were sampled at 50 kHz after low-pass Bessel filtering at 10 kHz. Compensation circuitry was used to reduce the series resistance by at least 70%. To record the activation of VGSCs, voltage steps from −80 to +80 mV in 10 mV step increments were applied prior to a test pulse of −90 mV. Fast inactivation was measured at 0 mV after prior test pulses of 300 ms duration ranging from −100 to 0 mV in 10 mV voltage step increments after maintaining the holding potential at −90 mV during 10 ms.

### 2.4 Statistical Analysis

All data are expressed as means ± SEMs of *n* determinations. Data analysis was performed using GraphPad Prism 8. Statistical comparisons were performed using one-way analysis of variance (ANOVA), followed by post hoc Dunnett̀s tests. *p* values ≤ 0.05 were considered statistically significant, and IC_50_ values were determined by fitting the data with a log (inhibitor) *vs* normalized response model.

The combination index (CI) was calculated with the Chou–Talalay equation^33^ following the formula
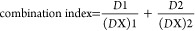
1where (*D*X)1 is the IC_50_ value of CTX3C alone, (*D*X)2 the IC_50_ value of BTX-3 alone, and *D*1 and *D*2 are the IC_50_ values of CTX3C and BTX-3, respectively, in combination. According to the Chou–Talalay method, additivity is established if CI = 1, synergism if CI < 1, and antagonism if CI > 1.

## 3 Results

Due to the co-occurrence in seafood products of BTXs and CTXs and the cellular target being the same, the sodium channels, in the present work, the single and combined effects of pacific ciguatoxin CTX3C and BTX-3 in human sodium channels were studied.

### 3.1 Effect of CTX3C or BTX-3 on Human VGSC

The effects of increasing CTX3C concentrations, between 0.000001 and 10 nM, on the maximum peak inward sodium currents (*I*_Na_) and the activation voltage of human VGSC after single cell exposure were first evaluated and reported by our group.^7^ As previously reported,^7,34,35^ CTX3C elicited a concentration-dependent decrease in the maximum peak amplitude of sodium currents being detected even at the lowest concentrations. In control conditions, the peak sodium current at −10 mV was −1156 ± 189 pA (*n* = 13) decreasing in a concentration-dependent manner up to −692 ± 372 pA (*n* = 3) after bath application of 1 nM CTX3C. The percent inhibition of the peak inward sodium currents by different CTX3C concentrations was used to obtain a concentration–response curve with an estimated IC_50_ of 0.173 nM [95% confidence interval (CI) from 0.000647 to 2.1 nM, *R*^2^ = 0.92]. CTX3C also affected the activation voltage of sodium channels and remarkably shifted in the negative direction after bath addition of 1 nM CTX3C as indicated by one-way ANOVA followed by Dunnett’s test. At this concentration, the negative shift in the activation voltage of sodium channels reached −15.9 ± 3.8 mV (*p* = 0.0004; d*f* = 21; *t* = 4.1). Higher toxin concentrations also hyperpolarized the activation voltage in a concentration-dependent manner, as summarized in Table 1.

**Table 1:** Activation Voltage of Human Na_v_1.6 VGSC after Cell Exposure to CTX3C, BTX-3, and a Combination of Increasing Concentrations of Both Marine Biotoxins

[CTX3C], nM	activation voltage for CTX3C (mV)	activation voltage for CTX3C + BTX-3 (mV)	activation voltage for BTX-3 (mV)	[BTX-3], nM
control	–30.7 ± 2.4	–30.0 ± 2.9	–36.2 ± 1.2	control
0.0001	–30.0 ± 0	–37.8 ± 3.2	–38.3 ± 1.1	0.1
0.001	–30.0 ± 0	–41.3 ± 2.3	–42.6 ± 1.0	1
0.01	–33.3 ± 3.3	–41.4 ± 1.4	–44.0 ± 1.6	5
0.1	–39.0 ± 1.8	–48.3 ± 4.8	–45.0 ± 2.2	10
1	–46.7 ± 2.9	–66 ± 4	–50.0 ± 0	50
5	–51.67 ± 4.8	–66.7 ± 3.3	–50.0 ± 0	100

The activity of BTX-3 in VGSC was also evaluated but, in this case, bath application of increasing BTX-3 concentrations, from 0.1 to 100 nM, did not cause any remarkable decrease in the maximum peak inward sodium current. The percent inhibition of VGSC amplitude (*I*_Na_) by the different BTX-3 concentrations was used to obtain a concentration–response curve with an estimated IC_50_ of 202 nM (95% CI: 55.3 to 6000 nM). A low variance value, very close to zero (0.23) was obtained, indicating that all the values were in a narrow range obtaining a low dispersion of the data since none of the concentrations elicited an important decrease in sodium current amplitude, with 100 nM BTX-3 decreasing peak sodium current amplitude only by 31.2 ± 15%. Noteworthily, at −20 mV, cell exposure to 1 nM BTX-3 did not elicit any significant decrease in the maximum *I*_Na_, which was −3696.2 ± 530.8 pA (*n* = 25) in control conditions and −2887.70 ± 402.0 pA in the presence of 1 nM BTX-3 (*n* = 23) which supposes a decrease of 21.9 ± 10.9%. However, cell exposure to the same toxin concentration significantly hyperpolarized the activation voltage of human VGSC by −7.3 ± 1.8 mV (*p* = 0.0003; *t* = 4.016, d*f* = 32). These results are in agreement with previous reports, where electrophysiological recordings showed that BTX-3 did not affect the amplitude of *I*_Na_ neither in protist sodium channels^5^ nor in other different cell lines,^36^ showing only a 33% decrease in peak inward sodium current in presence of 1000 nM BTXs in diatom sodium channels.^37^ Thus, the results obtained show that although CTXs and BTXs share their main site of action in VGSC, they trigger different functional effects, with CTX3C decreasing the maximum *I*_Na_ and hyperpolarizing the activation voltage of VGSC while BTX-3 only affected their activation voltage, shifting it toward more negative potentials, as summarized in Tables 1–3. Representative sodium channel activation traces at −20 mV in presence of 0.001 nM CTX3C, 1 nM BTX-3, and a combination of both are represented in Figure S1.

**Table 2:** Shift in the Activation Voltage of Human Na_v_1.6 Sodium Channels after Exposure to CTX3C, BTX-3, and a Combination of Increasing Concentrations of Both Marine Biotoxins

[CTX3C], nM	shift in the activation voltage for CTX3C (mV)	shift in the activation voltage for CTX3C + BTX-3 (mV)	shift in the activation voltage for BTX-3 (mV)	[BTX-3], nM
control	0	0	0	control
0.0001	0.71 ± 5.4	–7.78 ± 4.3	–2.07 ± 1.6	0.1
0.001	0.71 ± 4.7	–11.25 ± 3.7	–6.39 ± 1.5	1
0.01	–2.62 ± 5.6	–11.43 ± 3.5	–7.79 ± 1.9	5
0.1	–8.29 ± 3.3	–18.33 ± 5.2	–8.79 ± 2.7	10
1	–15.95 ± 3.8	–36.00 ± 4.9	–13.79 ± 2.6	50
5	–20.95 ± 4.8	–36.67 ± 5.4	–13.79 ± 3.6	100

**Table 3:** Normalized Maximum Peak of Sodium Currents after Cell Exposure to CTX3C Alone and CTX3C in the Presence of 10 nM BTX-3

[CTX3C], nM	normalized *I*_Na_ (max)	+10 nM BTX-3 normalized *I*_Na_ (max)
control	–1 ± 0.02	–1 ± 0.07
0.0001	–0.79 ± 0.05	–0.89 ± 0.08
0.001	–0.62 ± 0.08	–0.89 ± 0.13
0.01	–0.66 ± 0.09	–0.88 ± 0.13
0.1	–0.63 ± 0.09	–0.90 ± 0.11
1	–0.27 ± 0.07	–0.68 ± 0.09
5	–0.32 ± 0.04	–0.46 ± 0.09

### 3.2 Functional Consequences of the Combined Effects of CTX3C and BTX-3 on Human VGSC

In view of the results obtained separately for CTX3C and BTX-3 and the possibility of the simultaneous presence of both types of marine biotoxins in food, it is important to study the effects elicited by the presence of both marine compounds. The toxin concentrations evaluated for each compound were selected to cover a wide range of concentrations based on their individual effects in VGSC and the IC_50_ value obtained for each single compound. Therefore, cells were exposed to combined increasing concentrations of CTX3C from 0.0001 to 5 nM and BTX-3 from 0.1 to 100 nM.

**Figure 2:**
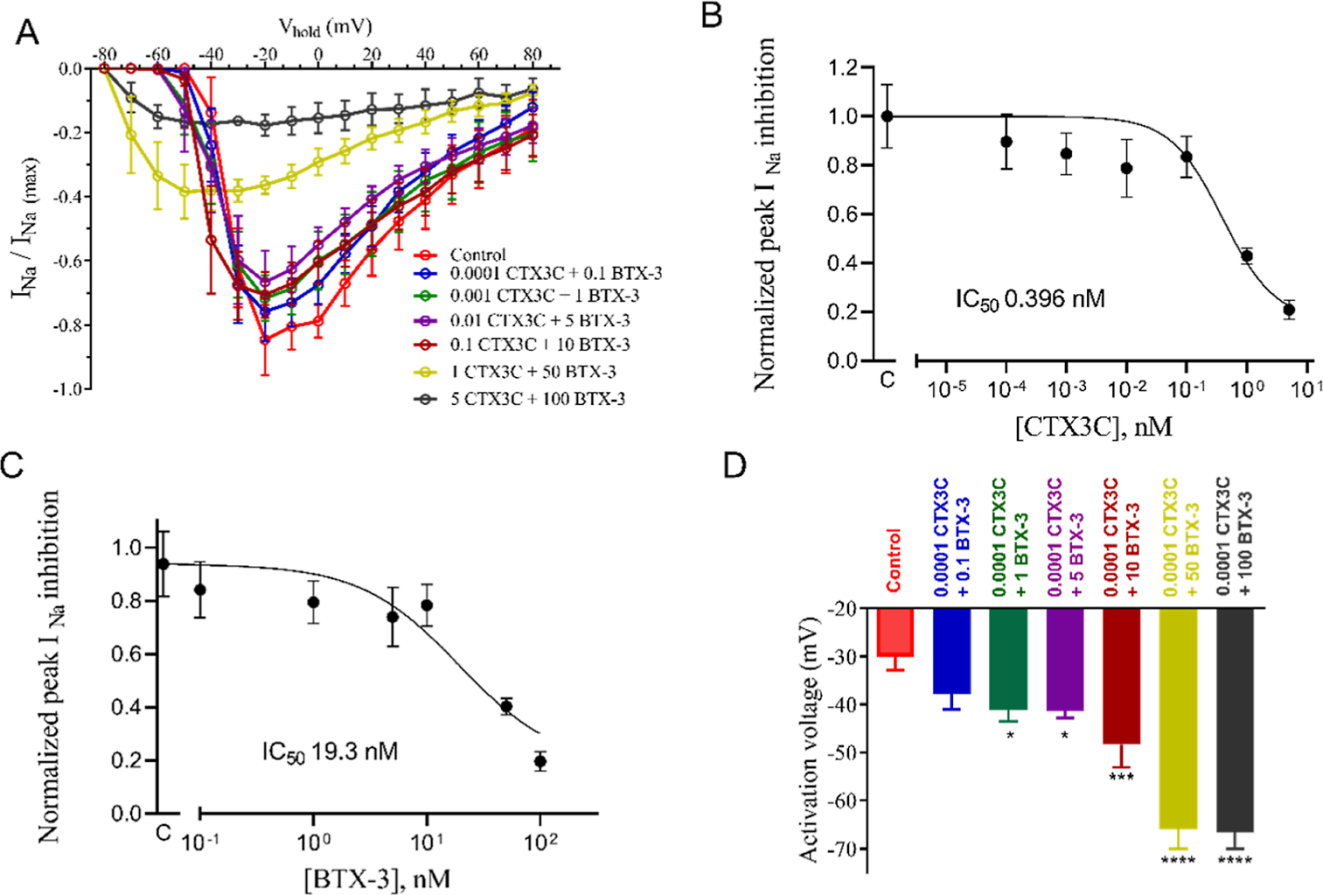
(A) Current–voltage relationship for the effect of different concentrations of combinations of CTX3C and BTX-3 on peak inward sodium currents. Toxin concentrations are expressed in nM. (B) Concentration–response graph for the peak inhibition of sodium currents by different CTX3C and BTX-3 concentrations relative to the CTX3C presence in the recording chamber. (C) Concentration–response graph for the peak inhibition of sodium currents by the simultaneous presence of CTX3C and BTX-3 expressed as a function of the BTX-3 concentration in the recording chamber. (D) Activation voltage of sodium channels in control conditions and after bath application of simultaneous increasing of BTX-3 and CTX3C concentrations, expressed in nM. *p < 0.05; **p < 0.01; ***p < 0.001 *vs* control currents.

The addition of combined increasing concentrations of CTX3C (from 0.0001 to 5 nM) and BTX-3 (from 0.1 to 100 nM) elicited a concentration-dependent decrease in sodium current amplitude and a remarkable hyperpolarization of the activation voltage, as shown in Figure 2A. The percent inhibition of the peak *I*_Na_ by the simultaneous addition of BTX-3 and CTX3C to the bath solution allowed to obtain a concentration–response curve as a function of the concentrations of each compound. Nonlinear fit of these data yielded an estimated IC_50_ based on CTX3C concentrations when combined with BTX-3 of 0.396 nM (95% CI: 0.0243 to 3.97 nM, *R*^2^ = 0.85), represented in Figure 2B, while the IC_50_ calculated taking into account the different BTX-3 concentrations in the presence of CTX3C was 19.3 nM (95% CI: 9.37 to 39.2 nM, *R*^2^ = 0.90), as illustrated in Figure 2C.

The activation voltage has been reported to be more sensitive to detect the effects of VGSC modulators;^11^ therefore, this parameter was evaluated to obtain the combination index of both marine biotoxins. In this case, the effect observed was very pronounced, as shown in Figure 2D. A significant negative shift of the activation voltage was obtained after cell exposure to concentrations of CTX3C as low as 0.001 nM; that alone did not elicit any change, with 1 nM BTX-3 hyperpolarizing the activation voltage by −6.39 ± 1.5 mV by itself, while the combination produced a hyperpolarization of the activation voltage of −11.25 ± 3.7 mV. The activation voltages in each condition are shown in Tables 1 and 2.

In summary, the concomitant increase of the concentrations of both marine biotoxins in the bath chambers shifted the VGSC activation voltage to more negative potentials, as shown in Table 2, which summarizes the changes in the activation voltage of human VGSC caused either by CTX or BTX alone or the combination of both compounds.

### 3.3 Functional Consequences Elicited by a Constant Low CTX3C Concentration and Increasing BTX-3 Concentrations on Human VGSC

In view of the results obtained over VGSC after cell exposure to different CTX3C and BTX3 combinations and to further explore their predominant effects, the cells were exposed to a constant CTX3C or BTX-3 concentration for 5 min in the recording chamber and increasing concentrations of the other toxin were added.

#### 3.3.1 Evaluation of the Effect of a Single Low Concentration of CTX3C with Increasing BTX-3 Concentrations on Human VGSC

**Figure 3:**
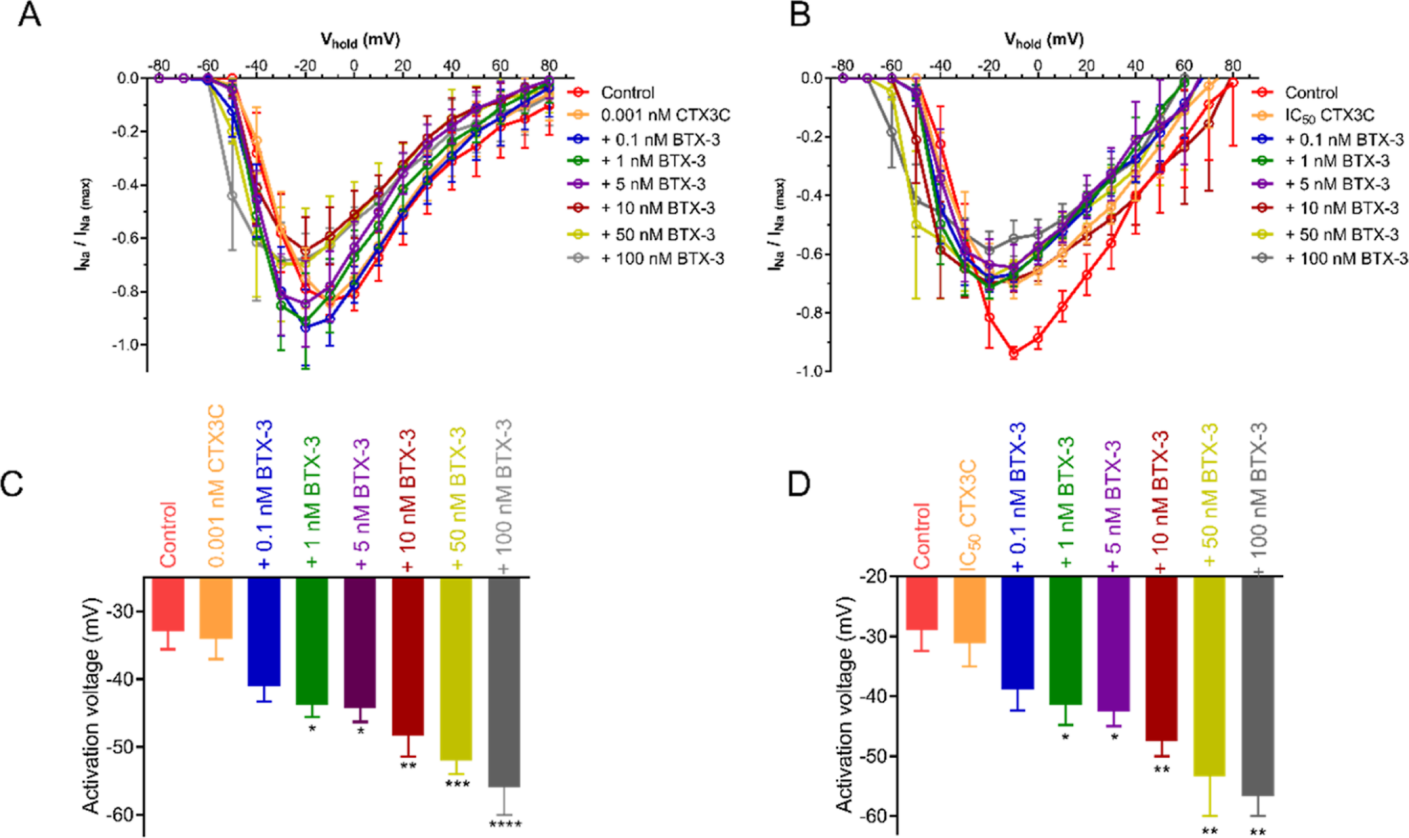
(A) Current–voltage relationship for the effect of CTX3C at 0.001 nM and different BTX-3 concentrations in *I*_Na_. (B) Current–voltage relationship for the effect of 0.17 nM CTX3C (concentration that corresponds to its IC_50_ in VGSC) and different BTX-3 concentrations in *I*_Na_. (C) Activation voltage of sodium channels in control conditions and after bath application of 0.001 nM CTX3C with increasing BTX-3 concentrations. (D) Bar graph showing the activation voltage of sodium channels in control conditions and after bath application of 0.17 nM CTX3C with increasing BTX-3 concentrations. *p < 0.05; **p < 0.01; ***p < 0.001 *vs* control currents.

Previous studies have reported a higher affinity of CTX3C to sodium channels than for BTXs;^8,9,14^ therefore, the next step was to study the effect of a constant low CTX3C concentration and progressively increasing the concentration of BTX-3 in the recording chamber. First, CTX3C at 0.001 nM was tested in combination with increasing BTX-3 concentrations. These data were compared with the effects of a constant CTX3C concentration equal to its IC_50_ concentration in the presence of different BTX-3 concentrations. As shown in Figure 3A, the addition of 0.001 nM CTX3C and increasing BTX-3 did not elicit any change in *I*_Na._ However, in cells exposed to 0.17 nM CTX3C, a decrease of the maximum *I*_Na_ peak was elicited, but it was not enhanced by the presence of further increasing BTX-3 concentrations, as represented in Figure 3B. In addition, the activation voltage of VGSC in these conditions was analyzed. As shown in Figure 3C, cell exposure to CTX3C at 0.001 nM did not modify the activation voltage of VGSC when added alone to the recording chamber, but its effect was enhanced after the addition of BTX-3 at concentrations of 1 nM and higher. Similarly, as shown in Figure 3D, when cells were exposed to 0.17 nM CTX3C, no changes in the activation voltage were observed; however, bath application of BTX-3 at concentrations of 1 nM and higher caused a negative shift in their activation voltage. These effects are summarized in Table 2.

#### 3.3.2 Evaluation of the Effect of a Single BTX-3 Concentration with Increasing CTX3C Concentrations in Human VGSC

**Figure 4:**
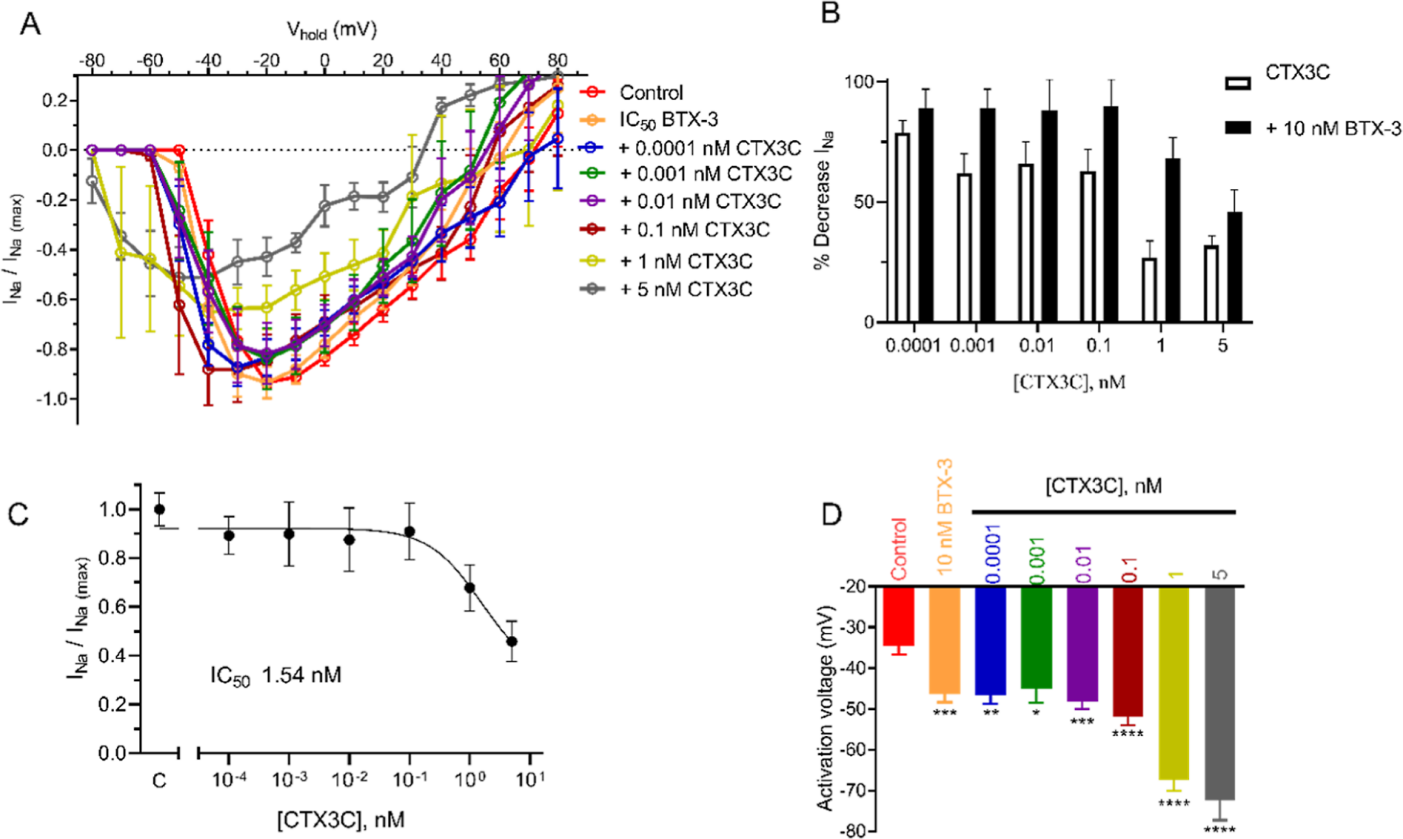
(A) Current–voltage relationship for the effect of 10 nM BTX-3 and increasing CTX3C concentrations on sodium current amplitude. (B) Percent decrease of *I*_Na_ caused by BTX-3 alone and the simultaneous presence of increasing CTX3C concentrations. (C) Concentration-curve graph for the peak inhibition of sodium currents by different CTX3C concentrations in the presence of 10 nM BTX-3 in the recording chamber. (D) Activation voltage of VGSC in control conditions and after bath application of 10 nM BTX-3 with increasing CTX3C concentrations. *p < 0.05; **p < 0.01; ***p < 0.001 *vs* control currents.

Previous studies have reported that CTX3C was able to displace BTX-3 from site 5;^14^ therefore, the effect of CTX3C in sodium channels was evaluated in the presence of an active BTX-3 concentration of 10 nM. BTX-3 alone hyperpolarized the activation voltage of human VGSC by −8.79 ± 2.7 mV, but higher CTX3C concentrations were needed to decrease peak sodium currents, as represented in Figure 4A,B. Thus, the presence of BTX-3 in the bath decreased the effect of CTX3C alone in *I*_Na_, as summarized in Table 3.

Nonlinear fit of these data yielded an estimated IC_50_ for CTX3C in the presence of 10 nM BTX-3 of 1.54 nM (95% CI: 0.797 to 3.03 nM, *R*^2^ = 0.95), represented in Figure 4C. As expected, the activation voltage of the sodium channels was significantly affected by the presence of 10 nM BTX-3 alone, but this effect was exacerbated after addition of different CTX3C concentrations to the recording chamber, as shown in Figure 4D.

**Figure 5:**
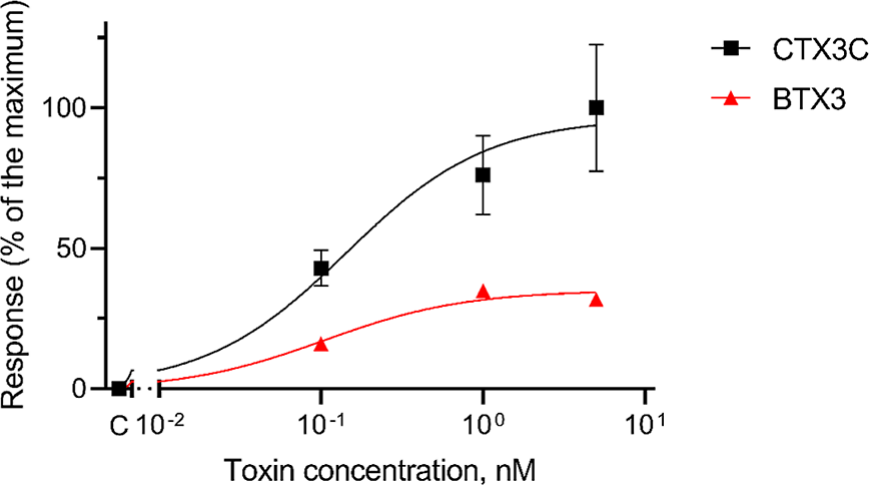
Graphical representation of the profile of the percentage of response on the negative change of the activation voltage of human VGSC produced by the full agonist CTX3C (black) and the partial agonist BTX-3 (red) added in the same concentrations to the bath chamber.

The negative displacement in the activation voltage of VGSC indicated that CTX3C acted as a full agonist of the channels, while BTX-3 was a partial agonist. This fact is demonstrated by the observation that a high concentration of BTX-3 caused a response typical of a competitive antagonist decreasing the effect observed with the full agonist alone. However, increasing the concentration of CTX3C displaced the partial agonist from their binding site and led to the decrease in *I*_Na_ caused by CTX3C alone. These results are illustrated in Figure 5.

### 3.4 Combined Effects of BTX-3 and CTX3C on Sodium Channel Activation: Combination Index

**Figure 6:**
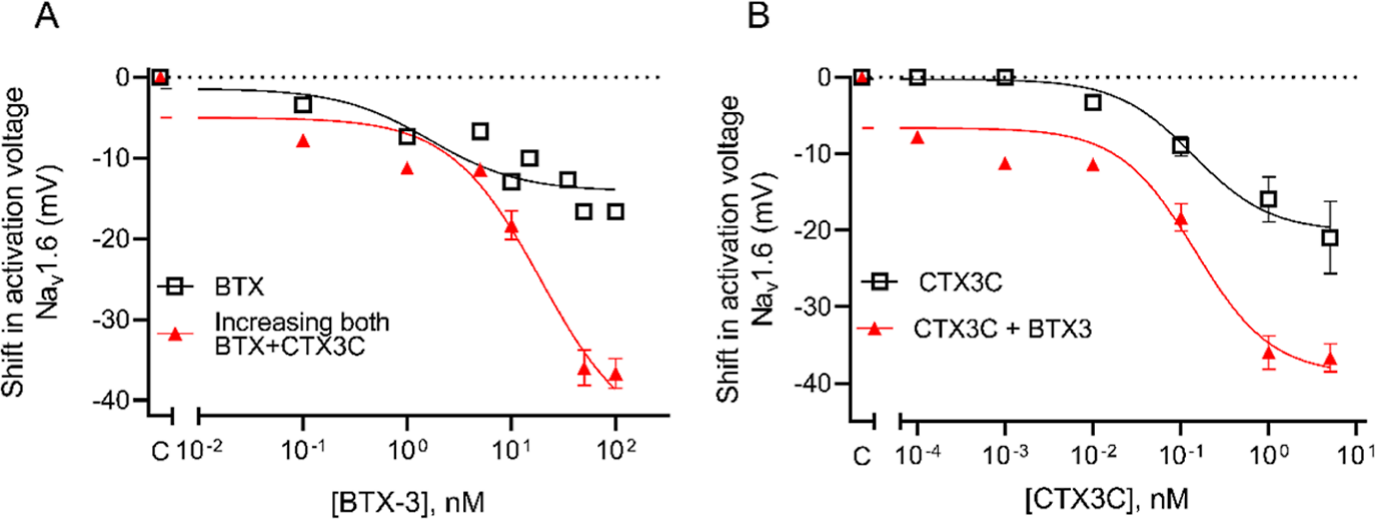
Single and combined effects of different CTX3C and BTX-3 concentrations in the activation voltage of human Na_v_1.6 VGSC. (A) Concentration–response graph for the negative shift in the activation voltage in different conditions represented against the BTX-3 concentrations in the bath chamber. (B) Concentration–response graph for the negative shift of the activation voltage in different conditions as a function of the CTX3C concentrations added to the cells.

The results obtained in this study, as previously described for CTXs alone,^11^ led to the conclusion that the change in VGSC activation could be the most sensitive parameter to analyze their functional effects in sodium channels. Figure 6 summarizes the effect of cell exposure to the different BTX-3 and CTX3C concentrations employed. These values of the activation voltage of human VGSC caused by CTX3C, BTX-3, and the combination of both were used to obtain the concentration–response curves. Nonlinear fit of the data yielded an estimated IC_50_ of 46.2 nM (95% CI: 35.5 to 60.8 nM) in the basis of the bath concentration of BTX-3 and an IC_50_ of 7.45 nM (95% CI: from 5.39 to 1.03 nM) for the simultaneous presence of CTX3C and BTX-3 based on BTX-3 concentrations, represented in Figure 6A as black and red lines respectively. Similarly, an IC_50_ for CTX3C of 2.58 nM (95% CI: 1.78 to 3.79 nM) and 0.195 nM (95% CI: 0.078 to 0.184 nM) was found for the simultaneous presence of CTX3C and BTX-3 based on CTX3C concentrations (Figure 6B). With these values, the combination index was determined and yielded a value of 0.24. This value is lower than 1, which indicates that the effect of these two marine biotoxins in the activation voltage of human VGSC is synergistic.

### 3.5 BTX, CTX, and Their Combinatory Effects on the Inactivation State of Human VGSC

**Figure 7:**
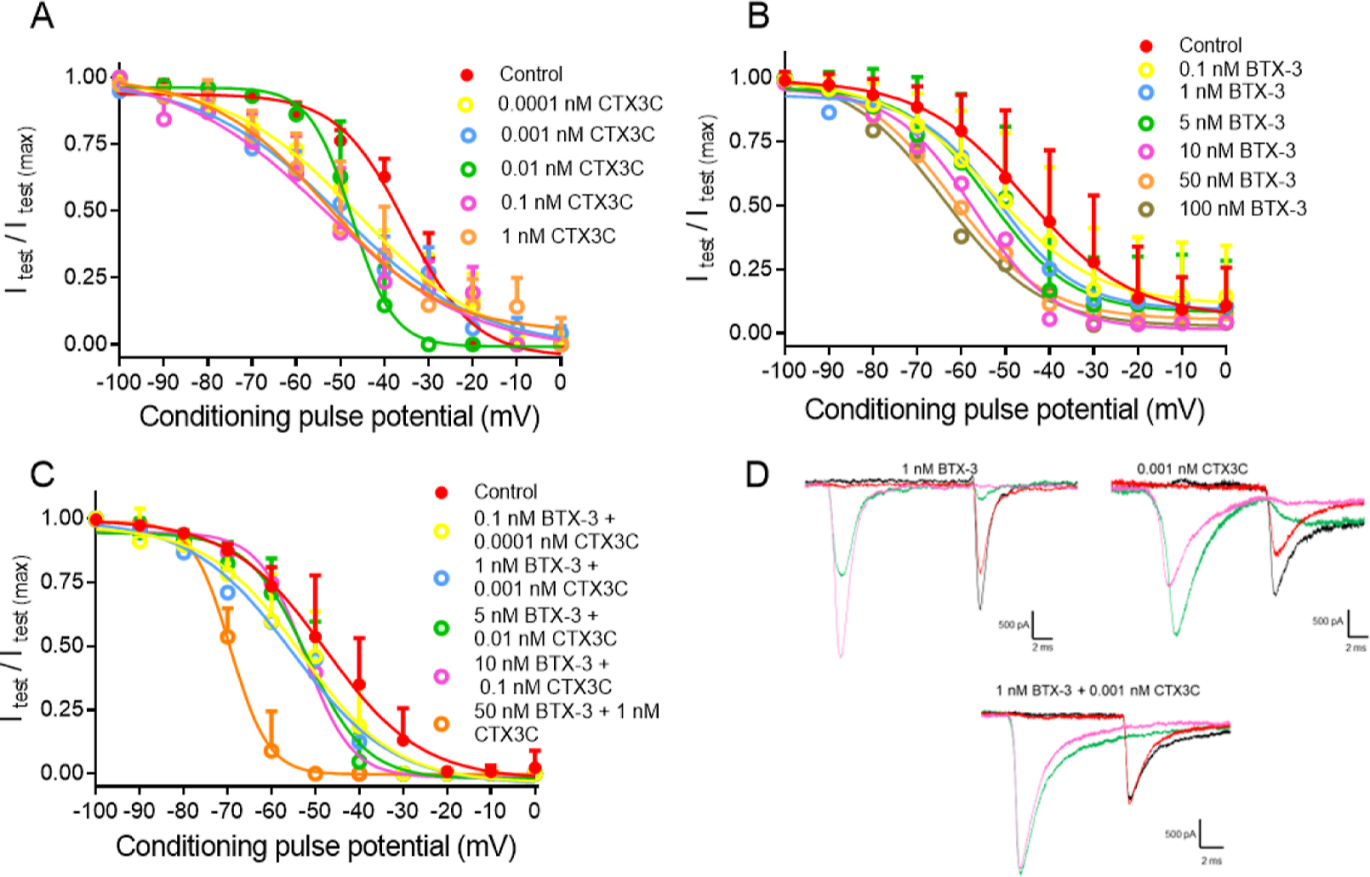
Single and combined effect on the fast inactivation of human VGSC of different CTX3C and BTX-3 concentrations and combinations. (A) Effect of CTX3C on Na_v_1.6 VGSC inactivation. (B) Effect of BTX-3 over the fast inactivation of Na_v_1.6 VGSC inactivation. (C) Effect of different CTX3C and BTX-3 combinations on the fast inactivation of human sodium channels. (D) Sodium current inactivation traces under different treatment conditions obtained at a prepulse potential of −60 mV (black for control and red for treated traces) and a prepulse of −30 mV in control (green traces) and treated cells (pink traces).

BTX-3 inhibits the fast inactivation of VGSC by its interaction with multiple active centers of the protein, specifically the A-ring lactone and the C-42 of the R side chain interacting with site 5 of the channel.^15,16^ Similarly, CTX3C shifts the half inactivation voltage (*V*_1/2_) in the negative direction.^38,39^ Thus, the effect of both compounds in the fast inactivation voltage of the channels was analyzed. As shown in Figure 7, BTX-3 elicited a significant change in the fast inactivation of human VGSC, *V*_1/2_ was −46.8 ± 1.8 mV (*n* = 20) in control conditions and −62.1 ± 3.9 mV (*n* = 18; *p* = 0.04) after bath application of 1 nM BTX-3. Cell exposure to higher toxin concentrations altered the inactivation state of VGSC, as shown in Figure 7A. In the same context, CTX3C shifted *V*_1/2_ of inactivation toward more negative potentials (Figure 7B) even at the lowest concentration studied. In control conditions, *V*_1/2_ of inactivation was −35.1 ± 1.2 mV (*n* = 7) and −45.8 ± 3.3 mV (*n* = 8) after bath application of CTX3C at 0.0001 nM (*p* = 0.0076). Thus, the combination of both marine biotoxins showed an enhanced effect on the fast inactivation state of human VGSC, as represented in Figure 7C and summarized in Table 4. A bar graph representation of the changes in *V*_1/2_ of human VGSC after cell exposure to all studied conditions are shown in Figure S2.

**Table 4:** Inactivation Voltage of Human VGSC After Cell Exposure to CTX3C, BTX-3, and a Combination of Increasing Concentrations of Both Marine Biotoxins

[CTX3C], nM	inactivation voltage for CTX3C (mV)	inactivation voltage for CTX3C + BTX-3 (mV)	inactivation voltage for BTX-3 (mV)	[BTX-3], nM
control	–36.1 ± 1.2	–41.7 ± 1.0	–45.1 ± 1.8	control
0.0001	–45.8 ± 3.3	–53.1 ± 1.7	–52.9 ± 2.1	0.1
0.001	–50.1 ± 4.1	–55.3 ± 2.2	–51.6 ± 2.1	1
0.01	–47.4 ± 1.4	–51.9 ± 1.2	–53.6 ± 2.8	5
0.1	–53.4 ± 6.8	–52.4 ± 0.9	–57.6 ± 2.4	10
1	–54.9 ± 5	–69.1 ± 0.4	–62.1 ± 3.9	50

The combination index for the effect of BTX-3 and CTX3C in the fast inactivation voltage of human VGSC was also calculated using the values of the hyperpolarizing shift in the inactivation voltage elicited by CTX3C, BTX-3, and the combination of both. Nonlinear fit of the data yielded an estimated IC_50_ for BTX-3 of 4700 nM (95% CI: 3300– 7800 nM) and 2500 nM (95% CI:11,580– 6260 nM) for the simultaneous presence of CTX3C and BTX-3 in function of the BTX-3 concentrations. An IC_50_ for CTX3C of 306 nM (95% CI:180– 900 nM) and 59.9 nM (95% CI: 33.8–247 nM) was obtained for the simultaneous presence of CTX3C taking into account the concentration of CTX3C. The combination index yielded a value of 0.73. This value is lower than 1 confirming a synergistic effect of CTX3C and BTX-3 on the inactivation state of human VGSC.

## 4 Discussion

The increasing expansion of marine biotoxins leads to a worldwide health concern that prompted public organizations to effectively evaluate the risks they pose to human health.^19,22,40^ CTXs and BTXs are two groups of compounds which share this characteristic, acting on the same cellular target, leading to similar neurological symptomatology in humans,^1,2^ and they cause similar *in vitro* effects. The combined effects of both food contaminants on sodium channel functionality have not been previously studied. CTXs decreased the maximum peak inward sodium currents at very low concentrations and cause a negative change in the activation voltage of up to 20 mV of VGSC, which is in accordance to previous reports^7,11,17^ and also their fast inactivation state.^35,38^ BTXs also caused an important negative displacement in the activation voltage of sodium channels of up to 13 mV not modifying the maximum *I*_Na_(4,5) and hyperpolarizing their fast inactivation voltage.^16^

The study of the functional effect elicited by the simultaneous presence of both marine toxins demonstrated that there is an enhancing effect on the negative shift of the activation voltage of human sodium channels when both BTXs and CTXs were combined. Therefore, a synergistic effect of both compounds in the activation voltage of human VGSC was demonstrated. The results presented here constitute the first report displaying an increased potency of CTX3C and BTX-3 on the activation voltage of human VGSC by 10 times after its combination. The consequence of the negative change in the activation voltage of VGSC triggered by both marine biotoxins in excitable cells is hyperexcitability.^41^ The results obtained for the fast inactivation state of sodium channels supported their synergistic effects and were in accordance with previous studies that found a shift of the *V*_1/2_ of inactivation by −15 or −18 mV^15,16,38,39^ in the negative direction. Since VGSC inactivation is a critical determinant of action potential frequency, defective inactivation is a hallmark of channel alterations, underscoring the critical importance of the inactivation function of VGSC in excitable cells.^42,43^ All these results explain the effects previously reported indicating spontaneous depolarization and oscillations of the membrane potential^17,35,44,45^ as well as the *in vivo* neurological symptomatology caused by these toxins.^1,2^ In summary, the results presented here indicate that CTXs and BTXs have a synergistic effect on human VGSC since the simultaneous exposure of the cells to a mixture of low concentrations of both compounds enhanced the effect elicited independently by each toxin on the activation as well as on the inactivation voltage of human VGSC. Therefore, the potentiation effect between BTXs and CTXs could pose a risk for human health, even at very low amounts since both compounds lead to neurological symptomatology that would be exacerbated by the concomitant presence of both seafood toxins.

The description of BTXs as partial agonists of the site 5 of VGSC has been previously reported indirectly in cerebellar granule cell neurons using calcium influx measurements.^46^ However, these experiments were performed in cells containing a wide array of receptors^47^ and in the presence of 0.04% pluronic acid that could alter cell membrane integrity,^48^ but no direct effect using electrophysiological methods has been reported so far. Therefore, the results presented in this paper constitute the first evidence of the synergic action of BTXs, acting as partial agonists of human VGSC, and CTX CTX3C as a full agonist of human VGSC. Despite acting on the same cellular target, the differences in the activity of both compounds on VGSC can be explained by the full agonist and partial agonist model. Remarkably, VGSCs undergo conformational changes reflecting their transition from resting to activated/open and to inactivated/closed states.^49^ In this paper, the functional interaction of BTXs and CTXs with VGSC was directly evaluated, and it was confirmed that the activation voltage of VGSC was more sensitive than the amplitude of sodium currents to detect the CTX and BTX groups of marine toxins.^11^

So far, the commonly used cell-based method for BTX and CTX detection was based on the evaluation of cell viability in the presence of ouabain and veratridine in mice neuroblastoma cells.^50^ However, N2a cells express a low number of VGSC with peak inward sodium currents between −200 and −400 pA.^51^ Therefore, the standardized protocol for CTX and BTX detection makes it difficult to establish a cell bioassay as a potential reference method for CTXs and BTXs.^52^ In this regard, the data presented here present the first approach to set up electrophysiology methods to evaluate the presence of marine toxins that can be automatized.^53^

There is one last subject that requires being clarified in further work. The synergistic interaction between CTXs and BTXs might suggest an allosteric effect that could be explained by two different binding sites at the same receptor that modify the binding energy of each toxin if the other is bound. We cannot provide a model with the evidence shown in this paper, but this would need further exploration.

## 5 Conclusions

There is a synergistic effect of the marine biotoxins CTX3C and BTX-3, potentiating their separate effect on VGSC, with both hyperpolarizing the activation voltage. It is also important to remark that the behavior of both compounds on VGSC is different since CTX3C acts as a full agonist, and BTX-3 act as partial agonist of human VGSC, being a determinant for the effect that they exert jointly and separately on the receptors. Noteworthily, for compounds that act on the same cellular target, it is important to determine the toxicity of each compound and their combinations at a wider level, both *in vivo* and *in vitro*, to establish safety limits for consumers.

## Supporting Information Available

Representative traces of sodium current activation recordings in human sodium channels and effect of different CTX3C and BTX-3 concentrations alone or the combinations of both toxins on their inactivation state (PDF)

## Abbreviations

BTXs: brevetoxins
CCFFP: Codex Committee on Fish and Fishery Products
CI: confidence interval
CP: ciguatera poisoning
CTXs: ciguatoxins
DMEM: Dulbecco’s Modified Eagle Medium
DMSO: dimethyl sulfoxide
EFSA: European Food Safety Authority
FDA: Food and Drug Administration
HEK: human embryonic kidney
*I*_Na_: VGSC current amplitude
mU: mouse units
nM: nanomolar
NSP: neurotoxic shellfish poisoning
*V*_hold_: holding potential
VGSC: voltage-gated sodium channels
