# Supplementary material for: Synergistic Effect of Brevetoxin BTX-3 and Ciguatoxin CTX3C in Human Voltage-Gated Nav1.6 Sodium Channels
Source: Chem Res Toxicol. 2023 Nov 15;36(12):1990–2000. doi: 10.1021/acs.chemrestox.3c00267 (PMC10845145; doi:10.1021/acs.chemrestox.3c00267)
Supplement: Supplementary file 1 — tx3c00267_si_001.pdf [file tx3c00267_si_001.pdf]

Supporting Information for:

Synergistic effect of brevetoxin BTX-3 and ciguatoxin CTX3C in human voltage gated Nav1.6 sodium channels

Sandra Raposo-Garcia†, Celia Costas†, M.Carmen Louzao†, Mercedes R. Vieytes‡, Carmen Vale†*, Luis M. Botana†*

†Departamento de Farmacología, Farmacia y Tecnología Farmacéutica, Facultad de Veterinaria, IDIS, Universidade de Santiago de Compostela, Campus Universitario s/n, 27002 Lugo, Spain.

‡Departamento de Fisiología, Facultad de Veterinaria, Universidad de Santiago de Compostela, Campus Universitario s/n, 27002. Lugo, Spain.

*Corresponding authors. E-mails: luis.botana@usc.es, mdelcarmen.vale@usc.es

Table of Contents

1. Figure S1……………..……………...................…..…..….…………………….….……...S2

2. Figure S2……………..……………...................…..…..….…………………….….…….…S3

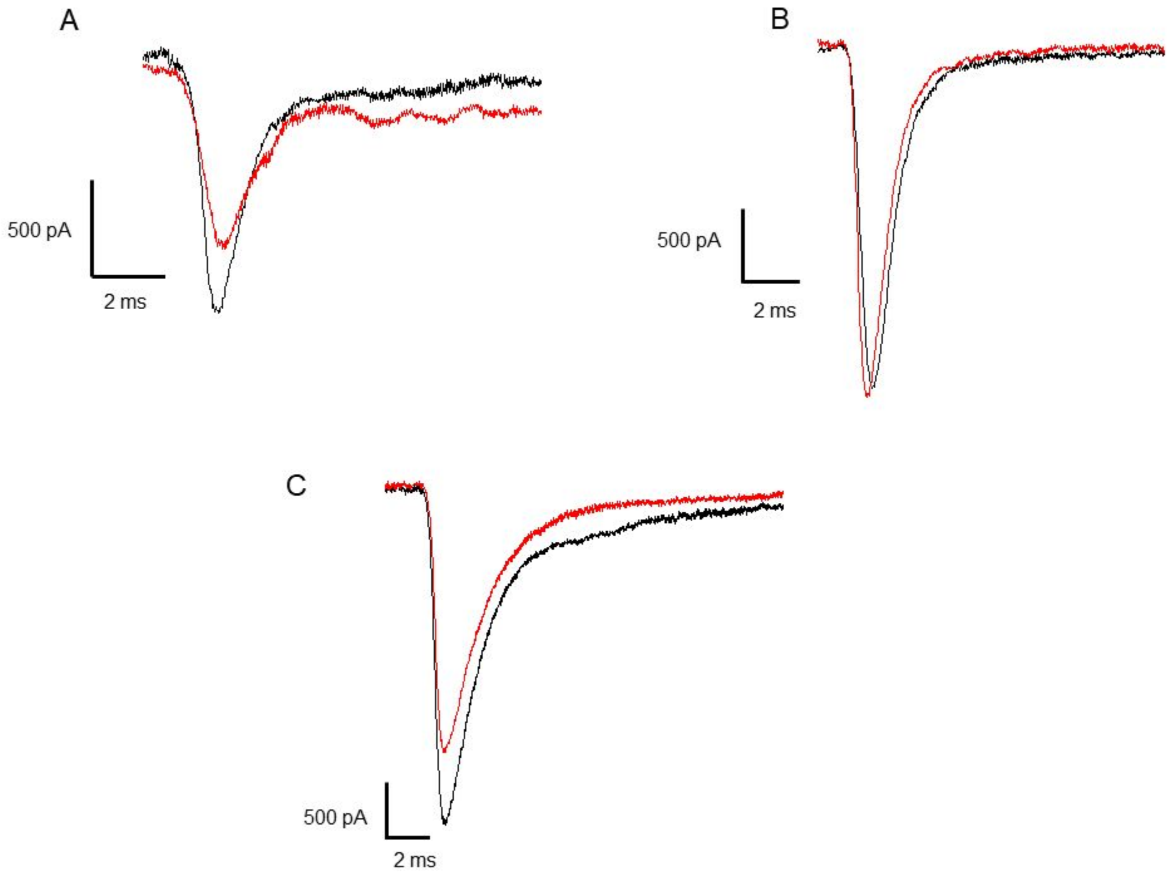

Figure S1: Representative traces of sodium current activation recordings at -20 mV in control conditions (black trace) and cells treated with A: 0.001 nM CTX3C. B: 1 nM BTX-3. C: 0.001 nM CTX3C and 1 nM BTX3.

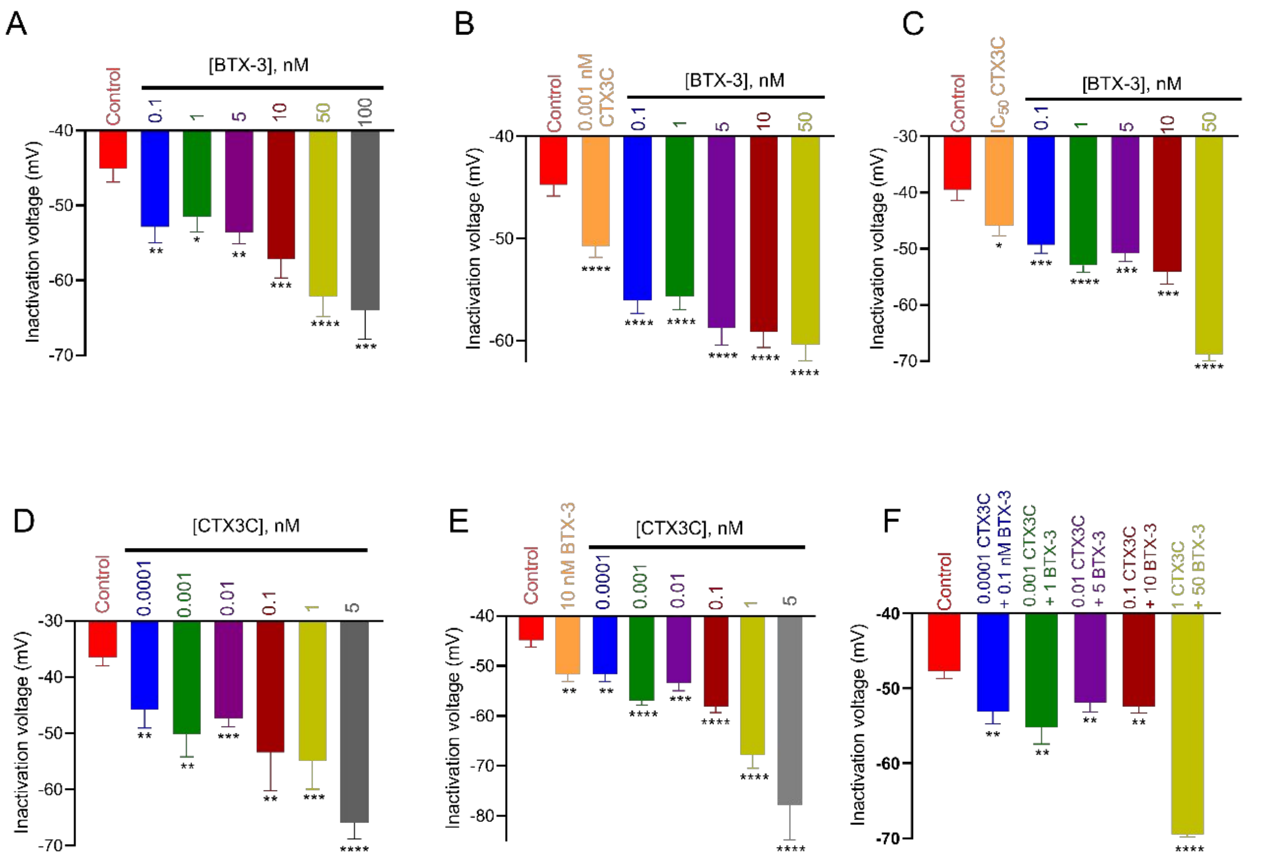

Figure S2: Single and combined effect on the inactivation state of human sodium channels of different CTX3C, BTX-3 concentrations and their combinations. **A**: Inactivation state of sodium channels in control conditions and after bath application of increasing BTX-3 concentrations. B: BTX-3 effect after cell exposure to 0.001 nM CTX3C. C: or an IC_{50} CTX3C concentration of 0.17 nM. **D:** Inactivation state of sodium channels in control conditions and after bath application of increasing CTX3C concentrations. E: CTX3C effect after cell exposure to 10 nM BTX-3. Inactivation state of human sodium channels in control conditions and after bath application of combined increasing BTX-3 and CTX3C concentrations, expressed in nM (**F**). * $p < 0.05$; ** $p < 0.01$; *** $p < 0.001$ vs control currents.
